# Genomic Analyses of *Globodera pallida*, A Quarantine Agricultural Pathogen in Idaho

**DOI:** 10.3390/pathogens10030363

**Published:** 2021-03-18

**Authors:** Sulochana K. Wasala, Dana K. Howe, Louise-Marie Dandurand, Inga A. Zasada, Dee R. Denver

**Affiliations:** 1 Department of Integrative Biology, Oregon State University, Corvallis, OR 97331, USA; howeda@oregonstate.edu (D.K.H.); denvedee@oregonstate.edu (D.R.D.); 2 Department of Entomology, Plant Pathology and Nematology, University of Idaho, Moscow, ID 83844, USA; lmd@uidaho.edu; 3 USDA-ARS Horticultural Crops Research Laboratory, Corvallis, OR 97331, USA; inga.zasada@usda.gov

**Keywords:** *Globodera pallida*, potato-cyst, nematode, plant-parasite, genome-wide genetic variation, *F_st_*, SNPs, allele frequency differentiation, genomics

## Abstract

*Globodera pallida* is among the most significant plant-parasitic nematodes worldwide, causing major damage to potato production. Since it was discovered in Idaho in 2006, eradication efforts have aimed to contain and eradicate *G. pallida* through phytosanitary action and soil fumigation. In this study, we investigated genome-wide patterns of *G. pallida* genetic variation across Idaho fields to evaluate whether the infestation resulted from a single or multiple introduction(s) and to investigate potential evolutionary responses since the time of infestation. A total of 53 *G. pallida* samples (~1,042,000 individuals) were collected and analyzed, representing five different fields in Idaho, a greenhouse population, and a field in Scotland that was used for external comparison. According to genome-wide allele frequency and fixation index (*F_st_*) analyses, most of the genetic variation was shared among the *G. pallida* populations in Idaho fields pre-fumigation, indicating that the infestation likely resulted from a single introduction. Temporal patterns of genome-wide polymorphisms involving (1) pre-fumigation field samples collected in 2007 and 2014 and (2) pre- and post-fumigation samples revealed nucleotide variants (SNPs, single-nucleotide polymorphisms) with significantly differentiated allele frequencies indicating genetic differentiation. This study provides insights into the genetic origins and adaptive potential of *G. pallida* invading new environments.

## 1. Introduction

As major agricultural pathogens, plant-parasitic nematodes cause numerous diseases in plants and are responsible for more than US$100 billion in annual agriculture loss worldwide [1,2]. Among the top three most significant plant-parasitic nematodes are the potato cyst nematodes *Globodera pallida* and *Globodera rostochiensis* [3]. Although these nematodes have a narrow host range that includes potato, tomato, and some solanaceous weeds, they can reduce potato yield by 80% [4,5]. Potato cyst nematodes are widely distributed in Europe; however, they are not widespread in North America [6]. Both species are considered quarantine pests in the U.S. *Globodera pallida* was first discovered in Idaho in April 2006 [7]. While infesting less than 1% of potato acreage in the state, the discovery of *G. pallida* in Idaho caused several countries to ban all fresh U.S. potato imports, though these markets have since been reopened [6,8]. Since the discovery of *G. pallida* in Idaho, containment and eradication of infested fields has been ongoing [6].

*Globodera pallida* initially evolved in South America, then was introduced into Europe, and from there spread to North America [9,10]. A spatial point pattern analysis suggests that the arrival and spread of *G. pallida* in Idaho resulted from a single introduction [11]. However, genetic evidence is lacking to evaluate whether *G. pallida* populations in Idaho stemmed from a single introduction or resulted from many. Knowledge on this is important from a regulatory perspective because population-level genetic variation is a key determinant of the strength of evolutionary adaptability; several introductions could lead to high within-species genetic diversity, which would increase the likelihood and pace of adaptation to the nematodes’ new environment.

*Globodera pallida* is a sedentary endo-parasite that undergoes sexual reproduction. Second-stage juveniles invade plant roots and establish feeding sites. As development continues, fertilized females enlarge to form cysts containing 200 to 600 eggs. Eggs require hatching factors, which are specific chemical cues released from host roots that induce hatching. In the absence of the host, eggs can remain dormant in the soil for many years [12]. Planting resistant crop varieties has historically been one of the most effective control measures for potato cyst nematodes; however, there are currently no russet potato varieties with resistance to *G. pallida* [13]. Crop rotation (with non-host crops) offers another control strategy; however, since some encysted eggs remain viable in soil for decades, even in the absence of a host crop, this strategy does not completely eradicate the nematode. Although no longer available for use in Idaho, the fumigant nematicide methyl bromide was initially used to eradicate *G. pallida*. Since *G. pallida* was discovered in Idaho in 2006, eradication efforts have aimed to contain and eradicate it through phytosanitary action and soil fumigation [6]. The effectiveness of eradication strategies depends on nematodes’ ability to adapt with changes in the environment and continue to survive. Therefore, knowledge on the potential for adaptation by nematodes to novel and changing environmental conditions is essential for crafting adaptive control strategies.

Studies of the genetic structure of populations provides important insights into the origins of invasive populations and their potential for adaptation to local and changing environmental conditions. Populations are generally thought to adaptively evolve through one of two main routes: wait for the appearance of a rare novel mutation which will sweep through the population if advantageous, or alternatively, through standing (i.e., pre-existing) genetic variation [14]. A third route would be migration which leads to new allele gains that will sweep through the population if advantageous [15]. Genome-wide next-generation sequencing of DNA pooled from multiple individuals is a highly effective way of locating genomic variation between wild populations [16]. Genome-wide studies facilitate the identification of candidate genomic regions harboring genes or genetic elements responsible for local adaptation by offering the ability to evaluate patterns of variation across different genomic regions [17]. Once a putative adaptive genetic variation related to the application of management practices is identified, alternative management strategies might be developed to specifically focus on eliminating the source of adaptive genetic variation. 

There are two main strategies aimed at identifying regions of the genome that may be responsible for local adaptation, particularly for non-model organisms [18,19]. The first strategy detects “outlier” loci (loci with unusually high genetic differentiation among populations) using the classic *F*-statistic/fixation (*F_st_*) index (differentiation outlier method). The second strategy tests the association between allele frequencies and environmental variables (genetic–environmental association method). 

*F_st_* is a widely used pairwise measure of population-level genetic differentiation and structure [20]. It is frequently estimated from DNA polymorphism data, such as single-nucleotide polymorphisms (SNPs). *F_st_* values range from zero to one; a zero value implies that the two populations under investigation are interbreeding freely and completely share genetic diversity, while a value of one implies complete isolation and an absence of shared genetic diversity. Local adaptation leads to strong genetic differentiation between populations, but only at specific genetic loci. Thus, loci with very high *F_st_* values compared to the rest of the genome are commonly identified to be under local adaptation and are referred to as outliers [18]. Genetic–environment association methods seek to identify alleles whose frequencies have strong correlations with environmental variable(s), which suggests the loci are involved in local adaptation [19]. Here, researchers hypothesize which environmental variable(s) are important in generating genetic variation that leads to local adaptation of wild populations.

In this study, a whole genome sequencing approach coupled with analysis strategies mentioned above were applied to Idaho *G. pallida* field populations to: (1) evaluate the genetic variation of *G. pallida* across fields to determine if the infestation was a result of a single or multiple introduction(s), and (2) explore temporal patterns of genome-wide polymorphisms to evaluate the adaptive potential of *G. pallida* populations in Idaho since the time of infestation.

## 2. Results

### 2.1. Sequence Yield and Read Mapping 

To compare patterns of genome-wide genetic variation among *G. pallida* populations from Idaho fields, the whole genomes from 53 cyst samples were sequenced; a population maintained in the greenhouse and a population from Scotland were also analyzed for comparative purposes. The yield of Illumina reads passing quality control measures varied from ~25 to 50 million reads for 52/53 samples (Supplementary Table S1). One sample, BIN25-7-d, had an unusually small number of quality reads numbering less than 3 million and was discarded. A total of 2.08 × 10^9^ quality filtered reads from the 53 samples were mapped to the *G. pallida* reference genome. All reads that did not match *G. pallida* were discarded. Although the quality filtered read yield did not dramatically vary among pre- and post-fumigation samples, the number of mapped reads to the *G. pallida* reference genome was low (<1.5 × 10^6^) in some of the post-fumigation samples (Supplementary Table S1). This observation was anticipated due to the visibly poor quality of encysted eggs from the post-fumigation samples, and only data from *G. pallida* cysts collected within one year post- fumigation (BIN25-1, BIN54-1, BIN258-1) were considered in subsequent analyses.

### 2.2. Effect of Coverage Threshold on Number of SNPs 

We identified SNP sites in our population-genomic data to assess genome-wide patterns of nucleotide variation among *G. pallida* populations. Each survey (Table 1) conducted to investigate *G. pallida* genetic differentiation across time and space was repeated with multiple minimum coverage thresholds (10X to 70X) to find the optimum minimum coverage threshold per survey. The total number of SNPs per survey decreased with increasing minimum coverage threshold (Table 2; Supplementary Figure S1). As expected, more stringent minimum coverage thresholds resulted in smaller numbers of observed SNPs. The minimum coverage applied to a particular survey was determined on a case-by-case basis, aiming to optimize the number of variable sites available for analysis while also maintaining high thresholds to avoid issues associated with low coverage; our parameters usually resulted in 100–300 SNPs available for surveys (Table 2). The optimum minimum coverage for surveys I and V-A was 30X and resulted in 198 and 255 SNPs, respectively. For surveys V-B and V-C, the optimum minimum coverage was 60X and resulted in 249 and 242 SNPs, respectively. It was 20X, 40X, and 70X for surveys II, III, and IV and resulted in 125, 100, and 302 SNPs, respectively. These SNPs were used to calculate SNP-specific *F_st_* values.

### 2.3. Genetic Differentiation Among G. pallida Field Populations in Idaho

**Figure 1:**
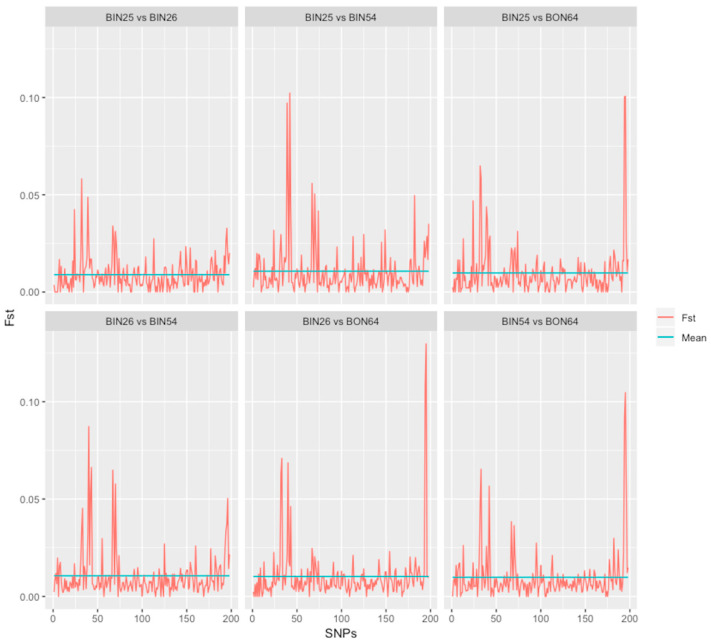
Genome-wide *F_st_* distribution between *Globodera pallida* populations obtained from four Idaho fields pre-fumigation. BIN25, BIN26, BIN54, BON64 designate individual fields, *F_st_*: fixation index. SNPs are arranged on the *x* axis by consecutive scaffold.

To assess large-scale patterns of spatial genetic differentiation among nematode populations in different Idaho fields, average *F_st_* was estimated for surveys I and II (Table 1). All the results presented here were obtained utilizing four biological replicates per field population. For survey I, in which comparison was made among pre-fumigation nematode populations collected in 2007 (BIN25, BIN26, BIN54, and BON64), a low *F_st_* average of 0.009 was obtained. To distinguish any population among these four fields with highly differentiated genomic regions, *F_st_* was calculated for every SNP site, and all possible pairwise population comparisons had low *F_st_* values, ranging from 0.0 to 0.13 (Figure 1). Next, SNP-specific allele frequencies were calculated and compared between these four fields to distinguish genomic regions with unusual genetic differentiation in any of these populations. For this analysis, 252 SNPs were obtained. When the Cochran–Mantel–Haenszel (CMH) test was implemented to assess significance of SNP-specific allele frequency differentiation among populations, no statistically significant differences were observed based on the standard genome-wide significance threshold of 5 × 10^−8^ [21]. 

**Figure 2:**
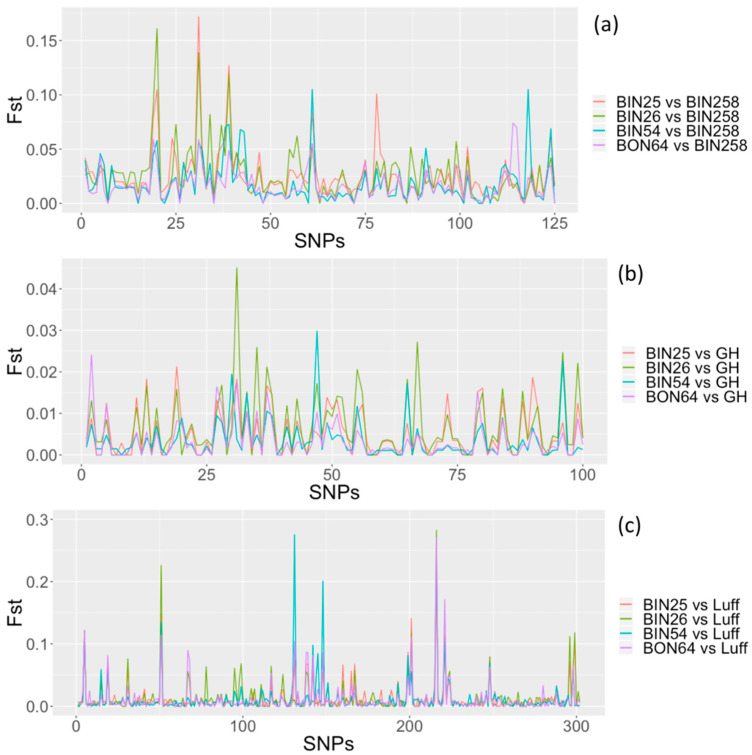
Genome-wide *F_st_* comparisons of *Globodera pallida* populations. (**a**) Comparison of BIN258 to other Idaho field populations, (**b**) comparison of greenhouse population to Idaho field populations, and (**c**) comparison of Scotland population to Idaho field populations. BIN25, BIN26, BIN54, BON64: designate Idaho fields, GH: Greenhouse, Luff: Scotland *G. pallida*. Colors represent *F_st_* distribution for all possible pairwise comparisons, *F_st_*: fixation index. SNPs are arranged on the *x* axis by consecutive scaffold.

When a field where infestation was more recently detected in 2014 (BIN258) was included in the analysis with the four 2007 fields (BIN25, BIN26, BIN54, and BON64 in 2007), an average *F_st_* of 0.013 was obtained (survey II). The genome-wide SNP-specific *F_st_* values calculated through pairwise population comparisons with BIN258 ranged from 0.0 to 0.175 (Figure 2a). For the SNP-specific allele frequency analysis, 62 SNPs were examined. Based on our CMH test results applied to SNP-specific allele frequencies, we found a total of twelve SNP sites with *p*-values that exceeded the standard genome-wide significance threshold (*p* < 5 × 10^−8^). Accordingly, we identified these twelve SNPs as significantly differentiated SNPs in BIN258 field population compared to the four field populations detected in 2007 (BIN25, BIN26, BIN54, and BON64). The twelve SNP outliers (SNPs with significantly differentiated allele frequencies) were located on two scaffolds of *G. pallida* genome: HG821129 and HG821413. The annotation results revealed genes (coding regions) associated with one of the SNP outliers. The single SNP outlier detected on scaffold HG821413 was associated with CLAVATA3/ESR (CLE-1) gene and identified as a non-synonymous mutation (amino acid replacement in the polypeptide). SNP outliers in the HG821129 scaffold (11/12 total) were placed in an intergenic region (non-coding) (Supplementary Table S2). 

### 2.4. Comparisons with Greenhouse and Scotland G. pallida Populations 

A *G. pallida* population maintained in a greenhouse at the University of Idaho, established from cysts collected from the BIN25 field population, was also included in the analysis for comparative purposes (survey III). *F_st_* analysis comparing samples obtained from field and greenhouse *G. pallida* populations had a low average *F_st_* of 0.009. All genome-wide SNP-specific *F_st_* values calculated through pairwise population comparisons with the greenhouse population were less than 0.05 (Figure 2b). For the SNP-specific allele frequency analysis, we investigated 29 SNPs and found no SNP-specific allele frequencies that were significantly differentiated between field and greenhouse *G. pallida* populations in Idaho (CMH statistical test, genome-wide significance threshold: 5 × 10^−8^). 

In order to provide an external context for our analyses, which were limited to Idaho field samples, a *G. pallida* population obtained from Scotland was used as an outside point of reference. When the Scotland population (Luff) was included in the analysis with the 2007 Idaho field populations (BIN25, BIN26, BIN54, and BON64), an average *F_st_* of 0.05 was obtained (survey IV). This was the largest average *F_st_* obtained compared to analyses including BIN258 (survey II: BIN25, BIN26, BIN54, BON64, BIN258) or greenhouse (survey III: BIN25, BIN26, BIN54, BON64, GH). In the analysis where *F_st_* was calculated for every SNP, the between population pairwise comparisons involving the Scotland *G. pallida* population had a genome-wide *F_st_* distribution that reached up to 0.3 (Figure 2c). The relatively high *F_st_* values indicated greater differentiation between the Idaho populations and the Scotland population (threshold *F_st_* for significant genetic differentiation = 0.25). The SNP-specific allele frequency analysis supported this observation with statistical evidence. From a total of 8296 SNPs, 1855 SNPs had *p*-values that exceeded the genome-wide significance threshold (*p* < 5 × 10^−8^, CMH test), indicating significant genetic differentiation between Idaho and Scotland *G. pallida* populations. These 1855 SNP sites were located on 257 scaffolds of *G. pallida* genome.

### 2.5. Patterns of Genome Wide Polymorphisms before and after Fumigation

**Figure 3:**
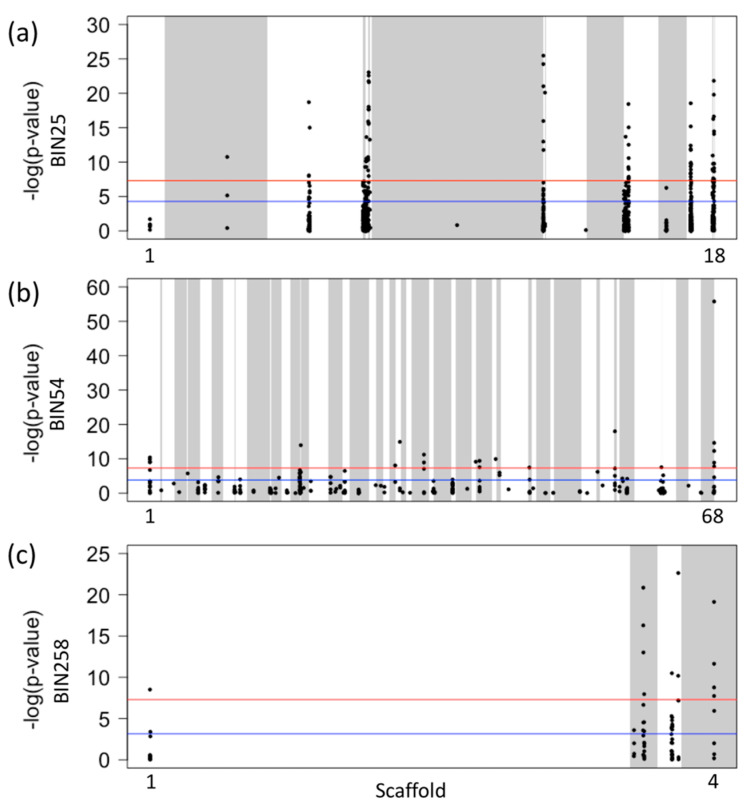
Evidence of significant allele (SNP) frequency change between pre- and post-fumigation samples across three *Globodera pallida* populations from Idaho. (**a**) BIN25, (**b**) BIN54, and (**c**) BIN258. The *y* axis indicates transformed *p*-values from the CMH statistical test. Bonferroni corrected significance threshold is shown in blue and standard genome-wide significance threshold is shown in red. The *x* axis indicates the genomic position; numbers indicate different scaffolds harboring SNPs. Shading of gray and white represent alternating scaffolds.

To assess temporal changes in genetic differentiation associated with fumigation, pre- and post-fumigation samples were evaluated from three *G. pallida* field populations: BIN25, BIN54, BIN258 (surveys V-A,B,C). All the results presented here were obtained utilizing four biological replicates per field and from two time points (24 total samples; 3 fields × 4 replicates × 2 time points). Genome-wide SNP-specific *F_st_* values calculated for pre- and post-fumigation sample comparisons provided evidence for significant genetic change in some regions in the genome over time. In order to provide an analytical context for pre–post comparisons, we used *F_st_* values resulting from pre–pre comparisons and post–post comparisons as references from each field. All genome-wide SNP-specific *F_st_* values resulted from comparison between BIN25 pre-fumigation samples (25-0 vs. 25-0) were less than 0.17. This included all possible pairwise comparisons between the four replicates of BIN25-0 (pre-fumigation). Similarly, comparisons between all BIN25 post-fumigation samples (25-1 vs. 25-1) had genome-wide SNP-specific *F_st_* values less than 0.12. However, the comparisons between BIN25 pre- and post-fumigation samples (25-0 vs. 25-1) had SNP-specific *F_st_* values that reached up to 0.35, indicating high genetic differentiation between pre- and post-fumigation samples obtained from this field based on our threshold *F_st_* of 0.25 (Supplementary Figure S2). Next, SNP-specific allele frequencies were calculated and compared between pre- and post-fumigation samples from BIN25 field. The SNP-specific allele frequency analysis supported the result from the *F_st_* analysis with statistical evidence. When the CMH test was implemented to assess the significance of SNP-specific allele frequency differentiation between BIN25 samples across time (pre- and post-fumigation), we found 73 sites (out of 956) with *p*-values that exceeded the standard genome-wide significance threshold (*p* < 5 × 10^−8^). These 73 SNP outliers (SNPs with significantly differentiated allele frequencies) were located on twelve scaffolds in the *G. pallida* genome resource (Figure 3a). The annotation results of these twelve scaffolds revealed five of them to have a total of 23 SNP outliers on protein coding genes. Some of these 23 SNP outliers were associated with ATP synthesis and protein folding functions, while others were associated with uncharacterized proteins. The SNP outlier on the ATP synthase gene was identified as a synonymous (silent) mutation. The rest of the genes resulted in both synonymous and non-synonymous mutations due to SNP outliers (Supplementary Table S2).

The comparisons between BIN54 pre-fumigation samples (54-0 vs. 54-0) had genome-wide SNP-specific *F_st_* values that were less than 0.03. Similarly, comparisons between BIN54 post-fumigation samples (54-1 vs. 54-1) had genome-wide SNP-specific *F_st_* values less than 0.02. But comparisons between BIN54 pre- and post-fumigation samples (54-0 vs. 54-1) had SNP-specific *F_st_* values as high as 0.24, which was closer to our threshold *F_st_* value of 0.25 (Supplementary Figure S2). When SNP-specific allele frequency analysis was performed to detect any genetic differentiation between pre- and post-fumigation samples obtained from this field, we found 22 (out of 329) significantly differentiated SNPs throughout the genome (*p* < 5 × 10^−8^, CMH test). These 22 SNP outliers were located on twelve scaffolds in the *G. pallida* genome (Figure 3b). Annotation results of these twelve scaffolds revealed one of them to have a SNP outlier on a protein coding gene that codes for Fido domain-containing protein. This SNP outlier was identified as a non-synonymous mutation leading to a change in the resulting polypeptide (Supplementary Table S2). 

All genome-wide SNP-specific *F_st_* values resulting from comparison between BIN258 pre-fumigation samples (258-0 vs. 258-0) were less than 0.03. Similarly, comparisons between BIN258 post-fumigation samples (258-1 vs. 258-1) had genome-wide SNP-specific *F_st_* values less than 0.07. The comparisons between BIN258 pre- and post-fumigation samples (258-0 vs. 258-1) had SNP-specific *F_st_* values that reached up to 0.16 (Supplementary Figure S2). To detect any genetic differentiation between pre- and post-fumigation samples obtained from this field, SNP-specific allele frequency analysis was performed and 12 SNP sites (out of 71) were detected with *p*-values that exceeded the standard genome-wide significance threshold (*p* < 5 × 10^−8^, CMH test). These twelve SNP outliers were located on four scaffolds in the *G. pallida* genome (Figure 3c). Annotation results of these four scaffolds revealed three of them to have outlier SNPs in protein coding genes. One SNP outlier was associated with protein folding function, while the other two were associated with uncharacterized proteins. While two of the SNP outliers on genes were identified as synonymous mutations, one resulted in a non-synonymous mutation that altered the resulting amino-acid (Supplementary Table S2). According to the SNP-specific allele frequency distribution patterns between pre- and post- fumigation samples, these polymorphisms (SNP outliers) were likely differentiated as a result of fumigation.

## 3. Discussion

Understanding the genetic structure of parasite populations and the process of genome evolution and adaptation is essential for crafting effective control strategies [22]. Accordingly, this study focused on a whole genome sequencing approach followed by population genetic analyses of a recent *G. pallida* infestation in Idaho. The first goal was to evaluate the genetic variation of *G. pallida* across Idaho fields to assess if the infestation was a result of single or multiple introduction(s). This is important from a regulatory perspective since several introductions could lead to high within-species genetic diversity, which would increase the likelihood and pace of adaptation to the nematodes’ new environment. Our assessment of population genetic structure using the *F_st_* index revealed patterns of genetic interrelatedness among *G. pallida* populations obtained from the Idaho fields. First considering the four fields, BIN25, BIN26, BIN54, and BON64, all 16 cyst samples from these four fields were collected in 2007 before fumigation began; therefore, any genetic effect that might occur from fumigation can be disregarded. The average *F_st_* for these four Idaho fields was estimated to be 0.009, which is very low. Wright suggested the following qualitative guidelines for the interpretation of *F_st_*: the range 0.0 to 0.05 indicates little genetic differentiation, the range 0.05 to 0.15 indicates moderate genetic differentiation, the range 0.15 to 0.25 indicates great genetic differentiation, and values above 0.25 indicate very great genetic differentiation [23]. Based on these guidelines, the average *F_st_* of 0.009 obtained for the four Idaho fields indicates close genetic relationships among the nematodes in these fields. 

However, Wright’s *F_st_* guidelines are general recommendations; *F_st_* is a relative rather than an absolute measure of genetic differentiation, and the value depends on the choice of populations or heterozygosity within populations. For example, *F_st_* values can be as low as 0.006 within one species, while that of another species can be as high as 0.714 [24]. For closely related mammalian species, typical values are of the order of 0.05 (5%) to 0.20 (20%), with average *F_st_* between human populations being 0.15 (15%) [25]. The animal-parasitic nematodes *Baylisascaris schroederi* had *F_st_* values ranging from 0.01 to 0.02 among different populations [26]. Therefore, to provide an external analytical context for our results, we compared the *G. pallida* field populations in Idaho with a *G. pallida* population obtained from Scotland and a population maintained in a greenhouse at the University of Idaho. When the Scotland population was added to the analysis with the Idaho fields (BIN25, BIN26, BIN54, BON64, and Luff), the average *F_st_* notably increased to 0.05. This is a 10-fold increase in relative terms and moderate genetic differentiation according to Wright’s general guidelines [23] between Idaho and Scotland populations. This result is consistent with a study done in Europe, where *G. pallida* populations obtained from the same area in France had *F_st_* values less than 0.1, while populations obtained from different European countries had *F_st_* values that showed a 10-fold increase and reached up to 0.45 [10]. Further, previous reports involving the plant parasite *G. rostochiensis* had *F_st_* values ranging from 0.02 to 0.40 among different populations [27]. When the greenhouse population was added to the analysis with the Idaho fields (BIN25, BIN26, BIN54, BON64, and GH), the average *F_st_* remained the same (0.009). This low *F_st_* value supports the finding that the *G. pallida* populations in Idaho fields and greenhouse are genetically similar in both relative and Wright’s terms. In fact, the greenhouse population was originally collected from the infested field BIN25 and maintained on susceptible potato (*Solanum tuberosum*) “Désirée” or “Russet Burbank”. The relative *F_st_* values confirmed close genetic relationships among the nematodes in Idaho fields. 

Our analysis on SNP-specific allele frequencies performed to detect any genomic regions with strong genetic differentiation among populations further supported the *F_st_* results. We found no statistically significant differences in SNP-specific allele frequencies among pre-fumigation *G. pallida* populations obtained from these four different Idaho fields. Further, we found no statistically significant differences in allele frequencies among Idaho field and greenhouse populations. However, analysis between Idaho and Scottish populations revealed 1855 SNP sites with significantly differentiated allele frequencies (*p* < 5 x 10^−8^), indicating greater genetic differentiation between the Idaho and Scotland populations. Moreover, we obtained consistent results for all biological replicates for all above analyses according to the CMH statistical test. Based on these results, we demonstrated that most of the genetic variation was shared among *G. pallida* populations from Idaho fields, indicating that the infestation likely resulted from a single introduction. It is common for plant-parasitic nematodes to gain entry to new areas through agricultural commodities (plant material or agricultural equipment contaminated with infested soil). After establishment and integration are achieved, mainly through local nematode reproduction, nematodes easily spread to nearby fields [11,28]. Therefore, *G. pallida* seems to have spread from field to field in Idaho depending on the introduction. Our results are consistent with a recent spatial pattern analysis of the *G. pallida* infestation in Idaho, which revealed that nearby infested fields contributed to the infestation of new fields through a contagion effect scenario [11].

Next, we compared fields where infestation was originally detected (in 2007) to a field where *G. pallida* was more recently detected in 2014 (BIN258). The cysts collected from BIN258 prior to fumigation were used for this analysis (similar to the four fields mentioned above). These five fields (BIN25, BIN26, BIN54, BON64, and BIN258) had a low average *F_st_* of 0.013, indicating very little genetic differentiation between them, similar to the results of the previous analysis. This suggests that BIN258 likely stemmed from the original infestation. However, SNP-specific allele frequencies analysis revealed twelve SNP sites with strong genetic differentiation in BIN258 compared to other Idaho fields (*p* < 5 × 10^−8^). Of the twelve outlier SNP sites observed in the analysis involving BIN258, eleven were found to be on non-coding regions of the genome. One outlier was associated with a CLE gene that is expressed in nematode parasitic stages, suggesting a role for their encoded proteins in plant parasitism [29]. This SNP outlier caused a non-synonymous mutation resulting in an amino acid change in the polypeptide, which might affect the protein function. The infestation in the BIN258 field was detected eight years after the initial *G. pallida* detection in Idaho. During this time period, the field had not undergone methyl bromide fumigation but was planted with potato. Thus, approximately seven generations are estimated to have occurred between 2007 and the sample collection at BIN258, assuming one generation per year/season based on the observation that cyst nematodes are monocyclic [30]. The detection of genomic loci with significantly differentiated allele frequencies in the BIN258 suggests that this Idaho field population has undergone local evolutionary adaptation, perhaps through mechanisms involving CLE genes, in the absence of eradication measures. Supporting our observations, experimental evolution studies on *G. pallida* and the model nematode *Caenorhabditis elegans* suggest that rapid and strong adaptation can happen in fewer than ten generations [31,32]. Further, Eoche-Bosy et al. have shown the feasibility of genome scan approaches to detect adaptation coming from short generation times [32].

Our next goal was to investigate patterns of genome-wide polymorphisms to evaluate the adaptive potential of *G. pallida* to soil fumigants. For this, 24 *G. pallida* samples were collected from three of the fields at two different time-points: (1) pre-fumigation and (2) the year immediately following fumigation (post-fumigation). This approach allowed us to look at potential genetic changes immediately surrounding fumigation treatments. If this genetic change is significant, it’s likely due to the survival of treatment resistant nematodes in post-fumigation populations, indicating potential for adaptation. Our analyses revealed higher genome-wide *F_st_* values between pre- and post-fumigation sample comparisons for the three fields evaluated (BIN25, BIN54, and BIN258), as compared to values between pre-fumigation comparisons and post-fumigation comparisons. This method provided the benefit of having a relative *F_st_* value to detect any genetic differentiation due to treatments, rather than having to rely on general *F_st_* guidelines. In certain regions of the genome, the SNP-specific *F_st_* values of pre–post comparisons relative to pre–pre and post–post comparisons, had a 10-fold difference and provided evidence for significant genetic change post-fumigation. Strong genetic differentiation like this due to contrasting habitats (non-fumigated vs fumigated) can be indicative of local adaptation that helps an organism to survive in a particular environment. To confirm local adaptation, one has to eliminate alternative explanations such as genetic drift. The *F_st_*, determined by genetic drift under selective neutrality, will affect all loci across the genome in a similar and predictable fashion [33], and this is not what we observed. Adaptation due to selection is a locus-specific force that can cause systematic deviations in *F_st_* values for a selected loci [33], and this is consistent with our SNP-specific *F_st_* results. 

To further support this observation, genome-wide SNP-specific allele frequencies were calculated and compared between pre- and post-fumigation samples. When statistical testing was applied to detect any statistically significant changes in SNP allele frequencies under more conservative thresholds (*p* < 5 × 10^−8^), 107 outlier SNP sites were found between pre- and post- fumigation samples across all three fields. This further supports the existence of genomic regions with strong genetic differentiation among pre- and post-fumigation populations. Strong outlier “signals” like these are taken to be indicative of local adaptation relative to background “noise” caused by migration, drift, or any other demographic changes that affect the whole genome equally [15,34]. Accordingly, we further analyzed these outlier SNPs to identify any genes associated with them. Among the 107 total outliers, 27 SNPs were located in protein coding genes associated with ATP synthase, protein folding, and potential parasitic functions (InterPro-EMBL-EBI). Among these 27 outliers in coding sequence, five SNP sites resulted in non-synonymous mutations that might affect the protein function. Understanding the potential functional effects associated with genes in which the outliers were detected requires further experimentation. Nonetheless, revealing the adaptive potential of invasive *G. pallida* provides important implications for its eradication, i.e., the eradication strategies could now focus on eliminating the source of adaptive genetic variation.

In conclusion, the patterns of genetic variation of *G. pallida* across Idaho fields suggest the infestation resulted from a single introduction, which acted as a point source for subsequent spread. *G. pallida* populations detected years after the initial detection indicate potential for local evolutionary adaptation in the absence of control measures. Temporal patterns of genome-wide polymorphisms involving pre- and post-fumigation samples revealed putative incipient adaptive alleles in *G. pallida* that might be caused by soil fumigants or other unknown stressors. Our genome-wide approach thus offered strong genetic insights into the origins of the Idaho *G. pallida* infestation and evidence of potential adaptive evolution in the decade following its initial introduction.

## 4. Materials and Methods

### 4.1. Globodera pallida Populations and Sampling

**Figure 4:**
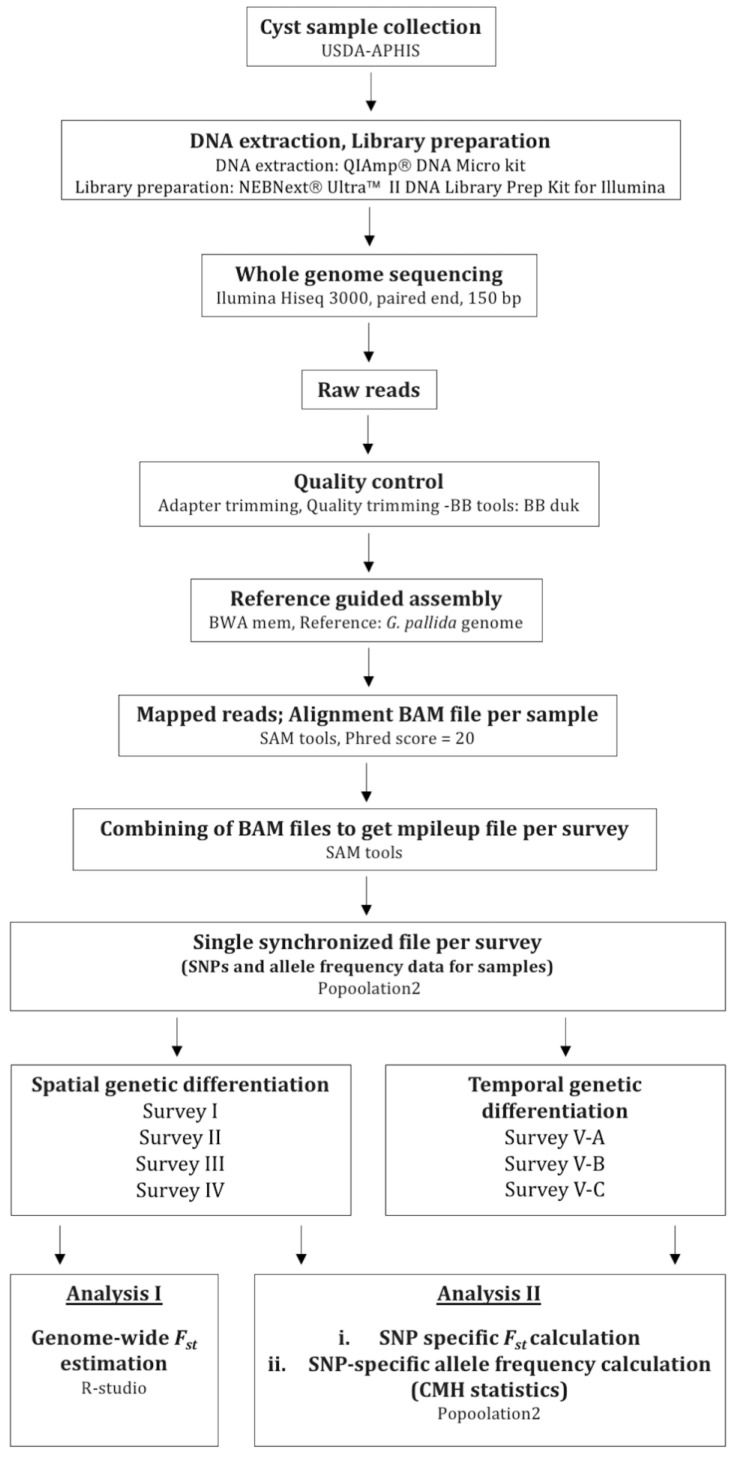
Workflow of data collection, bioinformatics and analysis methods. USDA-APHIS, United States Department of Agriculture, Animal and Plant Health Inspection Service; CMH, Cochran–Mantel–Haenszel test statistics.

**Figure 5:**
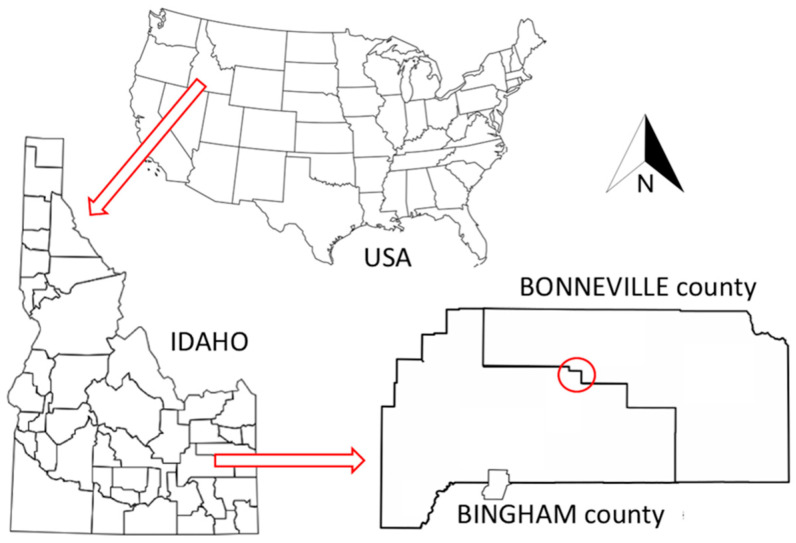
Location of fields in Idaho where *Globodera pallida* populations were collected.

The experimental approach began with the collection of *G. pallida* cysts from infested fields in Idaho (Figure 4). *G. pallida* cyst samples used in this study were originally collected between 2006 and 2014 by USDA-APHIS (United States Department of Agriculture, Animal and Plant Health Inspection Service) from fields in southern Idaho according to guideline protocols and using an agreed upon sampling protocol outlined by USDA-APHIS (https://www.aphis.usda.gov/plant_health/plant_pest_info/nematode/downloads/potato_guidelines.pdf (accessed on 18 March 2021)). The sampling method used by USDA-APHIS involved collecting 22.42 kg of soil samples per hectare, with cysts extracted from soil using the Fenwick flotation method [35]. Cysts were then stored dry until requested for this study. The identity of the original infestation of *G. pallida* was confirmed by morphological and molecular methods [36]. For the purpose of this study, a total of 53 samples altogether consisting of 2605 *G. pallida* cysts (~400 individuals per cyst) were processed. This included cyst samples from five different *G. pallida* infested fields in Idaho that were located within a 10 km radius in Bingham (BIN) and Bonneville (BON) counties (Figure 5). Cysts were collected from fields prior to fumigation; four cyst populations were collected in 2007, and one population was detected and collected in 2014. For each field population, four pooled samples (replicates) were collected. Each replicate consisted of 50 cysts (total of 200 cysts; approximately 200 × 400 individuals per field). For three of these fields, cyst samples were collected at two different time-points: (1) pre-fumigation and (2) post-fumigation. For comparative purposes, we included in the analyses a greenhouse *G. pallida* population maintained on potato originally collected from an infested field (BIN25) in Idaho and a European population from Scotland (Luffness) (Table 3).

### 4.2. DNA Extraction, Library Preparation and Genome Sequencing

Samples consisting of 50 cysts (~20,000 individuals) were used for DNA extraction; fewer cysts (5 cysts; 2000 individuals) were used from the Scotland population due to limited availability. For DNA extraction, cysts of pooled samples were crushed for 2 min with a motorized micro pestle and processed with QIAampDNA Micro kit (Qiagen, Hilden, Germany) following the manufacturer’s protocol. Total genomic DNA was sheared for 50 s using a Diagenode Bioruptor Pico (Diagenode, Inc., Denville, NJ, USA) to obtain peak library fragment sizes of ~500 bp, and genomic libraries were prepared using the NEBNext_Ultra II DNA Library Prep Kit for Illumina (New England Biolabs, Ipswich, MA, USA) following the manufacturer’s instructions. Whole genome sequencing (pool-seq) was performed using Illumina HiSeq 3000 for 2 × 150 bp reads (paired-end) at the Center for Genome Research and Biocomputing, Oregon State University, Corvallis, OR. Raw reads are available from NCBI’s (National Center for Biotechnology Information) Sequence Read Archive (SRA) under BioProject number PRJNA679610. 

### 4.3. Genome Assembly and Quality Control 

After sequencing, raw reads were trimmed to remove adaptors and filtered for quality using bbduk-BBtools (base calls with Phred quality score <20 were excluded from read ends) (http://jgi.doe.gov/data-and-tools/bbtools (accessed on 18 March 2021)). Filtered reads were then mapped to the *G. pallida* reference genome (NCBI accession number GCA_00724045, Reference guided assembly) using BWA mem (Burrows-Wheeler Aligner, version 0.7.12-r1039) [37]. In order to remove possible sample contamination, a more conservative strategy was employed to only analyze the reads that matched the *G. pallida* reference genome, and all the reads that mapped ambiguously were removed using SAMtools (version 1.3) [38]. For the filtered reads (that matched the nematode genome), base calling accuracy was evaluated by the Phred quality score, which indicates the probability that a given base is called correctly by the sequencer. A Phred quality score of 20 was used as the threshold, which means that the base call accuracy was 99%. The calls that did not fulfill this requirement were discarded. Quality filtered reads were then used for variant (SNP) calling, allele frequency computation, and further genome-wide genetic analysis (Figure 4). 

### 4.4. SNP Identification and Population Genomic Analyses 

SNP calling and allele frequency computation (at every base in the reference genome) were performed for each replicate of every population using SAMtools (mpileup) [38]. Then, population genomic analyses were conducted comparatively between different fields and time points to: (1) identify any distinct genetic groups indicating several introductions of the nematode (spatial genetic differentiation) and (2) detect any significant genetic change over time, suggesting the potential for adaptation (temporal genetic differentiation). Four and three specific surveys were conducted to evaluate spatial and temporal genetic differentiation of Idaho *G. pallida* populations, respectively (Table 1). First, to assess broad patterns of spatial genetic differentiation among populations, average *F_st_* was estimated (for surveys I to IV) using the formula:

$$F_{st}=\frac{\left(H_{t}-H_{s}\right)}{H_{t}}$$

where: *H_t_* = total heterozygosity and *H_s_* = average heterozygosity within each population.

Heterozygosity was calculated using major and minor allele frequencies of the populations being analyzed. The polymorphic sites where coverage was between 30X and 200X with a minimum minor allele frequency of 2% across all populations were considered here, and all sites failing to meet these criteria were discarded [39]. This first analysis was not applied to temporal surveys involving pre- and post-fumigation samples because in those surveys we were interested in finding genomic regions with potential adaptation rather than investigating differences in overall population structure (Figure 4).

Next, to distinguish any genomic regions with unusual genetic differentiation among populations, highly differentiated SNPs were identified. This analysis was applied to both spatial and temporal surveys (I to V-C) and involved a two-step procedure (Figure 4). First, genome-wide SNP-specific *F_st_* values were calculated for all possible pairwise population comparisons. The pairwise *F_st_* values were calculated using POPOOLATION2 software package [40] with a maximum coverage of 200X. A series of varying *n*-fold minimum coverage thresholds (10X–70X) were applied for each survey in order to determine the optimal minimum coverage for each particular survey (Table 2). The calculated *F_st_* values were then compared to a *F_st_* threshold of 0.25, which indicates a significant genetic differentiation [23]. The *F_st_* values were also comparatively evaluated to identify any significant genetic differentiation between populations. For spatial surveys, *G. pallida* populations obtained from greenhouse and Scotland were used for this purpose. For temporal surveys involving pre- and post-fumigation populations, comparisons were made between pre–pre, post–post, and pre–post samples per field.

Second, genome-wide SNP-specific allele frequencies were calculated and compared between populations (both spatially and temporally). Here, SNPs were identified in each survey using identical filtering criteria, i.e., SNP sites where coverage was between 40X and 200X were considered, with a minimum minor allele frequency of 2% across all populations. All sites failing to meet these criteria were discarded. Since four independent measurements of the allele frequencies were obtained per population (biological replicates), Cochran–Mantel–Haenszel test statistics (CMH test) [41] were implemented using the POPOOLATION2 software package [40] to detect the significant and consistent changes in SNP allele frequencies between *G. pallida* populations. To identify highly differentiated SNPs, the Bonferroni-corrected significance threshold and more stringent standard genome-wide significance threshold of 5 × 10^−8^ [21] were used. Only the SNPs exceeding both thresholds were considered to be outliers.

### 4.5. SNP Outliers and Coding Regions of the Genome

The scaffolds with SNP outliers (SNPs with significantly differentiated allele frequencies) were further analyzed to identify coding regions which corresponded to significantly differentiated sites. The information in WormBase ParaSite database (Version:WBPS14 (WS271)) on annotated exons (coding regions/genes) of the *G. pallida* reference genome was used to convert the synchronized file of scaffolds into a gene-based file [40]. Once the coding regions corresponding to significantly differentiated sites were identified, the WormBase Blastx function was used to discover any amino acid change in the resulting polypeptide due to SNP outliers (UniProtKB-WormBase ParaSite).

## Figures and Tables

**Table 1:** Specific Surveys Performed to Investigate Genetic Differentiation Among *Globodera pallida* Populations.

Category	Specific Survey	Populations Analyzed
Spatial genetic differentiation	Genetic differentiation among *G. pallida* populations from infested fields prior to fumigation (Survey I)	BIN25-0 BIN26-0 BIN54-0 BON64-0
	Genetic differentiation among Idaho field populations detected in 2007 and 2014 (Survey II)	BIN25-0 BIN26-0 BIN54-0 BON64-0 BIN258-0
	Genetic differentiation among field and greenhouse-reared Idaho populations (Survey III)	BIN25-0 BIN26-0 BIN54-0 BON64-0 Greenhouse
	Genetic differentiation among Idaho and Scottish *G. pallida* populations (Survey IV)	BIN25-0 BIN26-0 BIN54-0 BON64-0 Luffness
Temporal genetic differentiation	Patterns of genome-wide polymorphisms of pre- and post-fumigation populations (Surveys V- A, B, C)	BIN25-0 BIN25-1
		BIN54-0 BIN54-1
		BIN258-0 BIN258-1

Populations are referred based on geographic origin and time collected; “-0” indicates pre-fumigation, and “-1” indicates post-fumigation. Each survey had four replicate samples per population.

**Table 2:** Effect of Minimum Coverage Threshold on Number of Single Nucleotide Polymorphisms (SNPs).

Populations	Number of Pairwise Comparisons	Minimum Coverage	Number of SNPs
Survey I: BIN25-0_BIN26-0_BIN54-0_BON64-0 (16)	120	30	198
		20	1246
		10	50,508
Survey II: BIN25-0_BIN26-0_BIN54-0_BON64-0_BIN258-0 (20)	190	30	6
		20	125
		10	16,890
Survey III: BIN25-0_BIN26-0_BIN54-0_BON64-0_GH (20)	190	40	100
		30	3361
		20	3361
Survey IV: BIN25-0_BIN26-0_BIN54-0_BON64-0_Luff (5)	15	70	302
		60	1313
		10	1,240,364
Survey V-A: BIN25-0 vs BIN25-1 (8)	28	30	255
		20	1008
		10	6076
Survey V-B: BIN54-0 vs BIN54-1 (8)	28	60	249
		50	552
		20	18,729
Survey V-C: BIN258-0 vs BIN258-1 (8)	28	60	242
		50	653
		20	17,941
		10	92,847

Each *Globodera pallida* population had four replicate samples and the number in parenthesis indicates the total number of samples due to replicates. Populations are referred based on geographic origin and time collected; “-0” indicates pre-fumigation, and “-1” indicates post-fumigation.

**Table 3:** *Globodera pallida* Samples Collected from Infested Fields in Idaho for DNA Extraction.

Infected Field	Time Point	Date of Cyst Collection	Number of Replicates
BIN25-0	pre-fumigation	May 2007	4
BIN25-1	post-fumigation-1	Aug 2007	4
BIN25-3	post-fumigation-3	Jun 2008	4
BIN25-7	post-fumigation-7	Apr 2010	4
BIN25-10	post-fumigation-10	Oct 2012	4
BIN26-0	pre-fumigation	May 2007	4
BIN54-0	pre-fumigation	Apr 2007	4
BIN54-1	post-fumigation-1	Jul 2007	4
BIN54-11	post-fumigation-11	Oct 2012	4
BON64-0	pre-fumigation	May 2007	4
BIN258-0	pre-fumigation	May 2014	4
BIN258-1	post-fumigation-1	Aug 2015	4
Greenhouse	Control	Jan 2017	4
Luffness	Sample from Scotland	-	1

Each replicate consisted of 50 cysts (~20,000 individuals), except for the single sample from Scotland, which consisted of 5 cysts (~2000 individuals). “-0” indicates pre-fumigation, and “-1, 3, 7, 10, 11” indicates post-fumigation cycle.

## Data Availability

The data generated in this study are openly available in NCBI’s Sequence Read Archive (SRA) under the BioProject number PRJNA679610.

## Supplementary Materials

The following are available online at https://www.mdpi.com/2076-0817/10/3/363/s1, Figure S1: Effect of minimum coverage thresholds on the number of single nucleotide polymorphisms (SNPs) in the comparison of two *Globodera pallida* field populations, Figure S2: Genome-wide *F_st_* distribution of *Globodera pallida* populations obtained from three Idaho fields pre- and post-fumigation, Table S1: Whole genome sequencing yields for 53 *Globodera pallida* populations, Table S2: Genome annotation results (UniProtKB-WormBase Parasite) for the *G. pallida* scaffolds with significant outliers based on CMH test applied to allele frequency differences.
