# Supplementary material for: Genomic Analyses of Globodera pallida, A Quarantine Agricultural Pathogen in Idaho
Source: Pathogens. 2021 Mar 18;10(3):363. doi: 10.3390/pathogens10030363 (PMC8002896; doi:10.3390/pathogens10030363)
Supplement: Supplementary file 1 [file pathogens-10-00363-s001.pdf]

Supplementary Materials

Table S1. Whole genome sequencing yields for 53 *Globodera pallida* populations. All samples were mapped to the *G. pallida* reference genome (GCA_00724045). Samples are referred based on geographic origin and time collected; “-0” indicates pre-fumigation and, “-1,-3, -7, -10, -11” indicates post-fumigation cycles.

**Sample**	**Number of QC-passed reads**	**Number of reads mapped to the reference genome**
GH-a	47,376,071	32,934,412
GH-b	49,455,054	21,620,641
GH-c	39,673,127	20,727,066
GH-d	32,163,333	14,327,450
BIN258-0-a	36,387,604	18,243,704
BIN258-0-b	42,863,387	18,826,422
BIN258-0-c	47,507,442	26,582,651
BIN258-0-d	32,516,698	16,839,586
Luffness	39,181,272	26,904,465
BON64-0-a	32,182,916	20,165,728
BON64-0-b	31,544,307	8,763,994
BON64-0-c	31,277,548	18,965,578
BON64-0-d	31,998,395	23,517,334
BIN54-0-a	43,382,044	33,869,660
BIN54-0-b	37,035,806	23,648,073
BIN54-0-c	34,174,420	25,303,349
BIN54-0-d	50,966,677	42,732,435
BIN26-0-a	46,664,014	7,284,177
BIN26-0-b	43,479,684	10,280,241
BIN26-0-c	51,288,855	12,996,166
BIN26-0-d	43,698,016	9,515,354
BIN25-0-a	32,391,545	6,557,342
BIN25-0-b	47,681,698	12,412,393
BIN25-0-c	28,538,669	6,403,689

BIN25-0-d	42,425,929	15,055,873
BIN25-1-a	42,771,740	2,797,548
BIN25-1-b	31,064,333	1,798,764
BIN25-1-c	39,901,953	7,499,911
BIN25-1-d	40,710,883	2,577,196
BIN25-3-a	24,108,685	923,918
BIN25-3-b	40,462,356	1,541,586
BIN25-3-c	30,310,737	865,576
BIN25-3-d	43,185,469	2,294,860
BIN25-7-a	30,092,478	480,366
BIN25-7-b	38,597,910	1,001,883
BIN25-7-c	36,463,184	834,005
BIN25-7-d	2,966,145	1,20,503
BIN25-10-a	40,391,865	1,319,136
BIN25-10-b	53,416,108	1,643,741
BIN25-10-c	52,087,677	1,166,449
BIN25-10-d	37,211,201	818,949
BIN54-1-a	49,123,070	11,719,804
BIN54-1-b	48,786,524	8,442,937
BIN54-1-c	40,492,626	7,079,375
BIN54-1-d	37,089,462	5,489,429
BIN54-11-a	44,481,783	1,542,767
BIN54-11-b	43,944,443	2,314,082
BIN54-11-c	55,814,729	3,342,234
BIN54-11-d	39,487,867	922,296
BIN258-1-a	41,050,918	16,127,368
BIN258-1-b	27,571,120	3,071,405
BIN258-1-c	32,417,315	5,084,536
BIN258-1-d	42,388,142	10,132,192

Table S2. Genome annotation results (UniProtKB - WormBase Parasite) for the *G. pallida* scaffolds with significant outliers based on CMH test applied to allele frequency differences. Shaded regions represent coding regions.

Survey	Scaffolds with outliers	Outlier positions	Coding/Intergenic	Annotation Based on UniProtKB	Mutation type (Silent or replacement)
Survey II<br>25-0<br>26-0<br>54-0<br>64-0<br>258-0	HG821129 (scaff_423)	46979, 46993, 47002, 47030, 47043, 47045, 47047, 47063, 47065, 47080, 47094	Intergenic region	N/A	N/A
	HG821413 (scaff_509)	49299 (G/C)	Protein coding gene<br>47,853-49,560<br>(525 bp)<br>(174 aa)	Exon 4<br>49194-49322<br>(129 bp)<br><br>CLAVATA3/ESR-like protein<br>(CLE-1 gene)<br>A0A0K0KDJ7	Replacement<br>E (Glu) to Q (Gln)
Survey V-A<br>25-0 vs 25-1	HG819337 (scaff_123)	122942 (C/T)	Protein coding gene<br>121782-123696<br>(1617 bp)<br>(538 aa)	Exon 5<br>122,848-123,332<br>(485 bp)<br><br>ATP synthase<br>A0A183BW50	Silent
	HG819528 (scaff_145)	81817 (C/T), 81819 (C/G), 81826 (C/G)	Protein coding gene<br>81,059-86,899<br>(2280 bp)<br>(759 aa)	Exon 1<br>81059-83291<br>(2233 bp)<br><br>Lon N-terminal domain-containing protein<br>(Protein folding)<br>A0A183BXS7	81817 & 81826 Silent<br><br>81819 Replacement<br>A (Ala) to G (Gly)

		83372		Intron (83372)	N/A
	HG820077 (scaff_2107)	5156	Intergenic region	N/A	N/A
	HG820126 (scaff_2177)	570, 1076, 1559, 1698, 1702, 5007, 5009, 5010, 5386	Intergenic region	N/A	N/A
	HG820432 (scaff_2655)	616, 627, 630, 631, 638, 643, 660, 675, 679, 1102, 2928	Intergenic region	N/A	N/A
	HG820605 (scaff_2955)	265 (C/T) 291 (T/G), 317 (A/T), 346 (T/C),	Protein coding gene 221-364 (144 bp) (47 aa)	Exon 1 221-364 (144 bp)<br><br>Uncharacterized protein A0A183CQB4	265, 291, 317, and 346 Silent<br><br>317 Replacement F (Phe) to I (Ile)
		158, 568	Intergenic Region	N/A	N/A
	HG821116 (scaff_4205)	646	Intergenic region	N/A	N/A
	HG821519 (scaff_543)	3728 (T/C)	Protein coding gene 3580-4580 (735 bp) (244 aa)	Exon 2 3656-3839 (184 bp)<br><br>Uncharacterized protein A0A183CE60	Silent
		9215, 9234, 9306, 9677, 9684, 9685,	Intergenic region	N/A	N/A

		9711, 9712, 9714			
	HG821934 (scaff_676)	7859 (C/G), 7952 (C/T), 7992 (A/C), 7994 (C/G), 7997 (C/G), 8018 (C/G), 8071 (A/T), 8075 (C/G), 8110 (C/G), 8111 (C/G), 8123 (G/T), 8210 (C/G), 8228 (C/G), 9169 (A/T)	Protein coding gene 7782-9347 (1566 bp) (521 aa)	Exon 1 7782-9347 (1566 bp)<br><br>Uncharacterized protein A0A183CGZ4	9169 Replacement Y (Tyr) to F (Phe)<br><br>Others Silent
	HG824472 (scaff_6011)	130, 590, 1102, 1108, 1118	Intergenic region	N/A	N/A
	HG824481 (scaff_6025)	352, 454	Intergenic region	N/A	N/A
	HG825369 (scaff_7921)	180, 190, 403, 667, 674, 910, 967, 1007, 1008	Intergenic region	N/A	N/A
Survey V-B 54-0 vs 54-1	HG819124 (scaff_100)	32879, 32881, 32883, 32886, 32894	Intergenic region	N/A	N/A
	HG820126 (scaff_2177)	4989	Intergenic region	N/A	N/A
	HG820729 (scaff_321)	99571	Intergenic region	N/A	N/A
	HG821031 (scaff_396)	69006	Intergenic region	N/A	N/A

	HG821115 (scaff_41)	212458, 212499	Intergenic region	N/A	N/A
	HG821293 (scaff_471)	16734 (A/T)	Protein coding gene 15964-16847 (411 bp) (136 aa)	Exon 2 174 bp (16674-16847)<br><br>Fido domain-containing protein (effector proteins) A0A183CCC9	Replacement I (Ile) to F (Phe)
	HG821319 (scaff_47)	61842, 61844	Intergenic region	N/A	N/A
	HG821334 (scaff_483)	59874	Intergenic region	N/A	N/A
	HG821454 (scaff_520)	26353	Intergenic region	N/A	N/A
	HG821854 (scaff_650)	16734	Intergenic region	N/A	N/A
	HG822472 (scaff_851)	9759	Protein coding gene 7822-11714 (1209 bp) (402 aa)	Intron	N/A
	HG825209 (scaff_7583)	41, 43, 49, 169, 185	Intergenic region	N/A	N/A
Survey V-C 258-0 vs 258-1	HG819528 (scaff_145)	81679 (G/A)	Protein coding gene 81,059-86,899 (2280 bp) (759 aa)	Exon 1 81059-83291 (2233 bp)<br><br>Lon N-terminal domain-containing protein (Protein folding) A0A183BXS7	Silent

	HG820077 (scaff_2107)	3121 (A/T)	Protein coding gene 3000-3694 (1437 bp) (478 aa)	Exon 9 2976-3152 (177 bp) Uncharacterized protein A0A183CP30	Silent
		2922, 2937, 2956		Intron	N/A
	HG820126 (scaff_2177)	3304 (G/A)	Protein coding gene 2501-3371 (669 bp) (222 aa)	Exon 4 3173-3371 (199 bp) Uncharacterized protein A0A183CP88	Replacement G (Gly) to D (Asp)
		4695, 4704	Intergenic	N/A	N/A
	HG821519 (scaff_543)	7224, 7229, 7232, 7237	Protein coding gene 5885-9155 (993 bp) (330 aa)	Intron	N/A

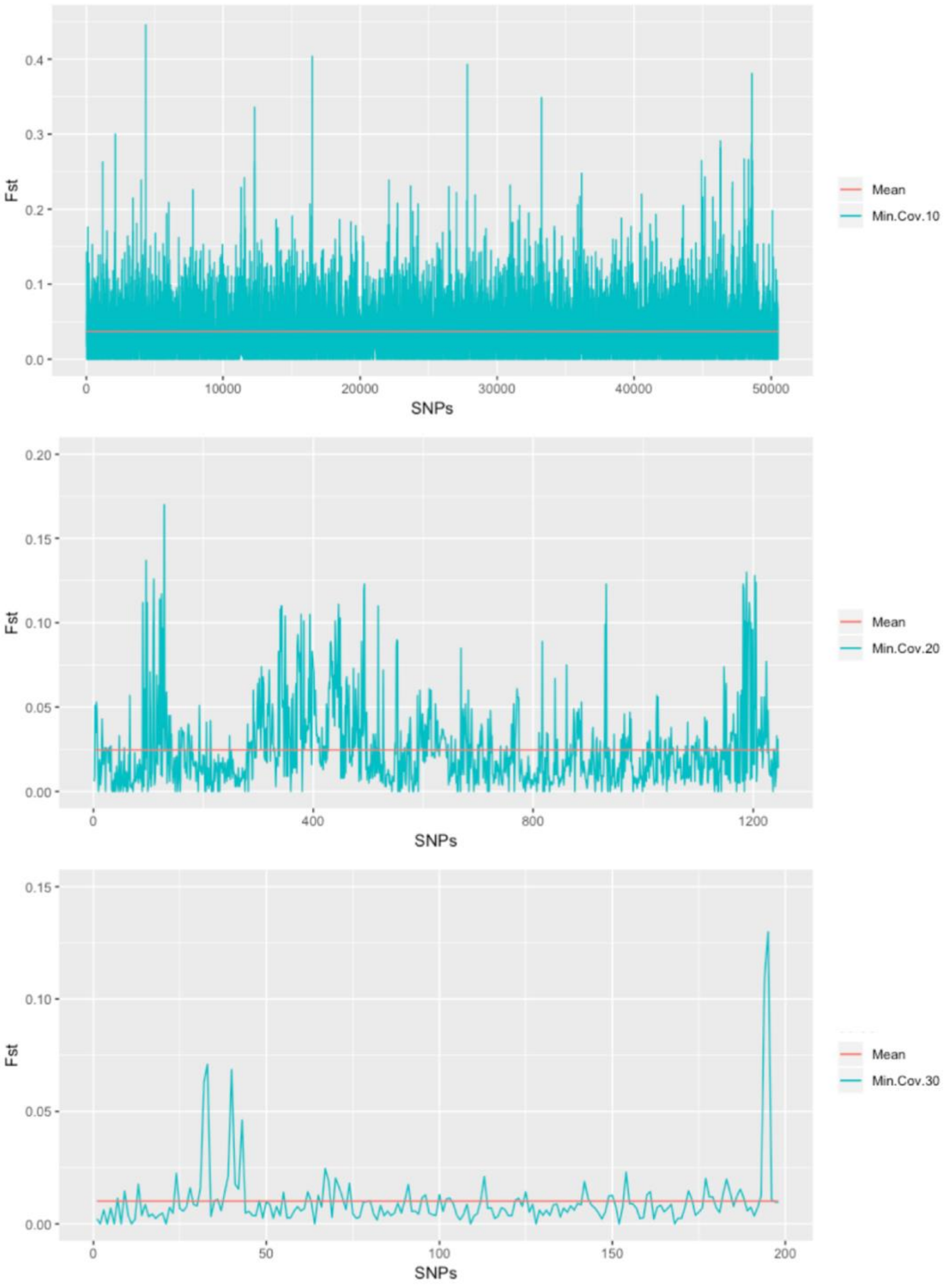

Figure S1. Effect of minimum coverage thresholds on the number of single nucleotide polymorphisms (SNPs) in the comparison of two *Globodera pallida* field populations (BIN26 and BON64).

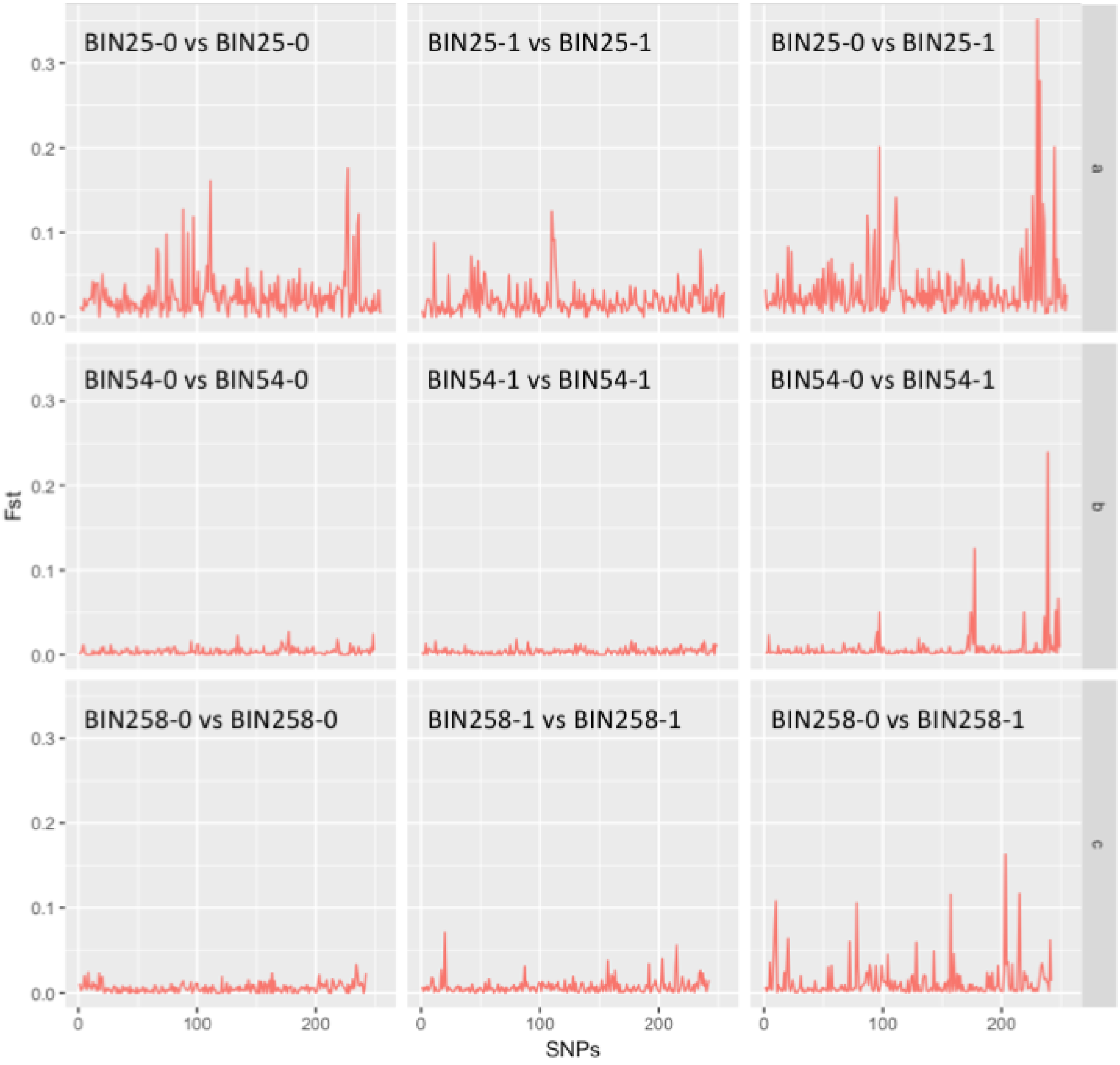

Figure S2. Genome-wide F_{st} distribution of *Globodera pallida* populations obtained from three Idaho fields pre- and post-fumigation. BIN25, BIN54, and BIN258: Idaho fields, "-0" indicates pre-fumigation and, "-1" indicates post-fumigation, F_{st}: Fixation index. SNPs are arranged on the X- axis by consecutive scaffold.
